# *CDH22* hypermethylation is an independent prognostic biomarker in breast cancer

**DOI:** 10.1186/s13148-016-0309-z

**Published:** 2017-01-24

**Authors:** Esperanza Martín-Sánchez, Saioa Mendaza, Ane Ulazia-Garmendia, Iñaki Monreal-Santesteban, Alicia Córdoba, Francisco Vicente-García, Idoia Blanco-Luquin, Susana De La Cruz, Ana Aramendia, David Guerrero-Setas

**Affiliations:** 1 Cancer Epigenetics Group, Navarrabiomed. Departmento de Salud-UPNA. IdiSNA, Irunlarrea Road, 3, 31008 Pamplona, Spain; 2 Department of Pathology, Complejo Hospitalario de Navarra, Irunlarrea Road, 3, 31008 Pamplona, Spain; 3 Department of Surgery, Complejo Hospitalario de Navarra, Pamplona, Spain; 4 Immunomodulation Group, Navarrabiomed. Departmento de Salud-UPNA. IdiSNA, Pamplona, Spain; 5 Department of Medical Oncology, Complejo Hospitalario de Navarra, Pamplona, Spain; 6 Biobank of Navarrabiomed. Departmento de Salud-UPNA. IdiSNA, Pamplona, Spain

**Keywords:** *CDH22*, DNA methylation, Breast cancer, Predictive biomarker

## Abstract

**Background:**

Cadherin-like protein 22 (CDH22) is a transmembrane glycoprotein involved in cell-cell adhesion and metastasis. Its role in cancer is controversial because it has been described as being upregulated in colorectal cancer, whereas it is downregulated in metastatic melanoma. However, its status in breast cancer (BC) is unknown. The purpose of our study was to determine the molecular status and clinical value of *CDH22* in BC.

**Results:**

We observed by immunohistochemistry that the level of CDH22 expression was lower in BC tissues than in their matched adjacent-to-tumour and non-neoplastic tissues from reduction mammoplasties. Since epigenetic alteration is one of the main causes of gene silencing, we analysed the hypermethylation of 3 CpG sites in the *CDH22* promoter by pyrosequencing in a series of 142 infiltrating duct BC cases. *CDH22* was found to be hypermethylated in tumoral tissues relative to non-neoplastic mammary tissues. Importantly, this epigenetic alteration was already present in adjacent-to-tumour tissues, although to a lesser extent than in tumoral samples. Furthermore, *CDH22* gene regulation was dynamically modulated in vitro by epigenetic drugs. Interestingly, *CDH22* hypermethylation in all 3 CpG sites simultaneously, but not expression, was significantly associated with shorter progression-free survival (*p =* 0.015) and overall survival (*p* = 0.021) in our patient series. Importantly, *CDH22* hypermethylation was an independent factor that predicts poor progression-free survival regardless of age and stage (*p* = 0.006).

**Conclusions:**

Our results are the first evidence that *CDH22* is hypermethylated in BC and that this alteration is an independent prognostic factor in BC. Thus, *CDH22* hypermethylation could be a potential biomarker of poor prognosis in BC.

**Electronic supplementary material:**

The online version of this article (doi:10.1186/s13148-016-0309-z) contains supplementary material, which is available to authorized users.

## Background

Breast cancer (BC) is the most frequent type of cancer among women and one of the leading causes of cancer-related deaths worldwide [1, 2]. In recent years, an increase in overall survival (OS) has been achieved, mainly due to advances in early detection programmes and therapeutic strategies, although its incidence remains high [2]. BC originates from the accumulation of genetic and epigenetic abnormalities in tumour suppressor genes and oncogenes [3]. A thorough understanding of the mechanisms responsible for BC onset and progression is needed to develop prognostic biomarkers and efficient targeted therapies.

BC comprises five major pathological subtypes: luminal A-like, luminal B-like (HER2-negative), luminal B-like (HER2-positive), HER2-positive (non-luminal) and triple-negative. This classification is based on immunohistochemical biomarkers (oestrogen, progesterone and HER2 receptors, and Ki-67), as confirmed in the last St Gallen International Expert Consensus [4]. However, these subtypes are heterogeneous and patients within a subtype can display a differential prognosis [5], so new prognostic biomarkers are still needed to stratify BC patients with good and poor outcomes [6].

Epigenetic alterations are common molecular abnormalities in cancer, including DNA methylation, alterations in microRNA profiling, and post-translational modifications of histones [7, 8]. Aberrant DNA methylation is one of the most frequent molecular abnormalities in BC [9]. Methylation of certain genes has been linked to clinical and pathological characteristics of breast tumours and is considered to be a biomarker of diagnosis [10], hormone receptor [11] and HER2 [12] status, response to tamoxifen [11] and chemotherapy [13], metastases during follow-up [9] and a predictor of survival [11, 14].

The *CDH22* gene, first described by Sugimoto et al. [15], is located on chromosome 20 and has 15 exons. It encodes a transmembrane glycoprotein of the cadherin family (known as CDH22 or PB-cadherin) that is involved in cell-cell adhesion. It has been found to participate in morphogenesis and tissue formation in neural and non-neural cells of the brain and neuroendocrine organs [16–18]. The expression of members of the cadherin family may affect tumorigenesis or metastasis of various cancers, and these proteins may serve as important biomarkers [16]. However, this gene has not been previously studied in BC. Our aim was to determine the molecular status and clinical value of *CDH22* in BC.

## Results

### CDH22 protein level is lower in BC tissues than in non-neoplastic tissues

**Fig. 1:**
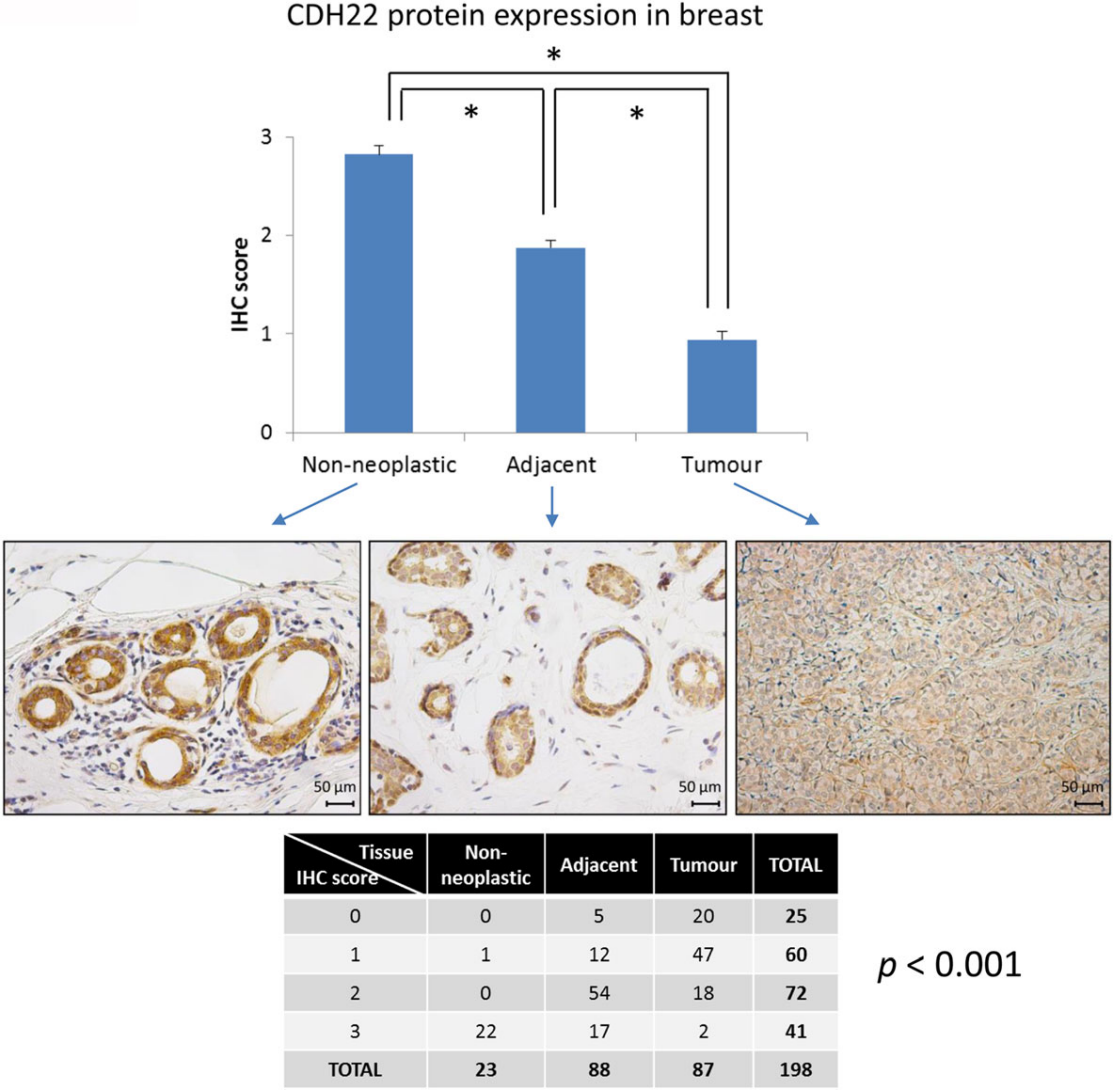
*CDH22* protein expression in breast tissues. CDH22 protein expression was measured by immunohistochemistry in 88 pairs of breast tumoural and adjacent-to-tumour tissues, along with 24 non-neoplastic samples from reduction mammoplasties. Expression levels were scored as 0, no expression; 1, weak expression; 2, intermediate expression; and 3, strong expression (*p < 0.001). Images were acquired using a Leica 4000B microscope (Leica, Wetzlar, Germany) at ×200 magnification. Contingency table shows the association between the tissue type and CDH22 immunohistochemical expression

In order to examine the CDH22 expression pattern in BC, we measured its protein level by immunohistochemistry in a series of 88 BC cases and their adjacent-to-tissue counterparts, along with 24 non-neoplastic samples from reduction mammoplasties. Overall, there was a significantly lower level of expression in tumour cells than in non-neoplastic cells (*p* < 0.001, Fig. 1). It is important to note that the adjacent-to-tumour tissue expressed an intermediate protein expression level, between the non-neoplastic and the tumour tissue. These results show for the first time the cytoplasmic protein expression pattern of CDH22 in BC and indicate that it is downregulated in this malignancy.

### The CDH22 gene promoter is hypermethylated in BC

Since DNA methylation is one of the main mechanisms of gene silencing, we investigated the methylation status of the *CDH22* gene. Five CpG sites in the *CDH22* promoter were examined by pyrosequencing in a larger series of 142 BC cases (Table 1), 26 paired adjacent-to-tumour tissues and 19 non-neoplastic breast samples from reduction mammoplasties. The *CDH22* promoter is enriched in poly-T sequences (Additional file 1: Figure S1), which makes it difficult to conduct successful pyrosequencing reactions of good quality. Especially, the presence of a poly-T very close to the second CpG introduced a large number of errors that hampered to analyse the methylation status of this second and subsequent CpG sites. To overcome this situation, two sequencing primers were used to gain coverage by sequencing more CpG sites in the region. Thus, methylation in CpG1 was analysed with a forward-sequencing primer, while CpG4 and CpG5 were examined with a reverse-sequencing primer (Additional file 2: Figure S2).

**Table 1:** Pathological and clinical characteristics of BC patient series

Variable	Frequency (%)
BC subtype	
LA	20/142 (14.1)
LB	44/142 (31.0)
LH	33/142 (23.2)
H	21/142 (14.8)
TN	24/142 (16.9)
Grade	
I	25/142 (17.6)
II	59/142 (41.5)
III	58/142 (40.8)
Lymph node involvement	
No	68/139 (48.9)
Yes	71/139 (51.1)
Stage	
I	49/138 (35.5)
IIA	34/138 (24.6)
IIB	27/138 (19.6)
IIIA	19/138 (13.7)
IIIC	9/138 (6.5)
Age (years)	Mean 60 Range 30–95
Tumour size (cm)	Mean 2.2 Range 0.3–10.0
Progression-free survival (months)	Mean 82.9 Range 1–208
No	115/141 (81.6)
Yes	26/141 (18.4)
Overall survival (months)	Mean 86.9 Range 1–208
Exitus	27/140 (19.3)
Chemotherapy	
No	49/138 (35.5)
Yes	89/138 (64.5)
Hormone therapy	
No	43/136 (31.6)
Yes	93/136 (68.4)

BC subtypes: *LA* luminal A, *LB* luminal B/HER2-negative, *LH* luminal B/HER2-positive, *H* HER2, *TN* triple-negative

Since pyrosequencing provides a quantitative measure of methylation, the optimal cut-off value distinguishing the unmethylated from the methylated status of each of the CpG sites was estimated by ROC curve analysis as being 17.5, 40 and 66.5% methylation for CpG1, CpG4 and CpG5, respectively, (Table 2 and Additional file 3: Figure S3). Additionally, we also considered that a case had hypermethylated *CDH22* when the three tested CpG sites simultaneously showed methylation percentages above their cut-off values.

**Table 2:** *CDH22* hypermethylation in our series of patients

Parameter	Number
Breast tumours	*n* = 142
Median % CpG1 methylation (range)	9.0 (1–100)
Median % CpG4 methylation (range)	58.0 (0–74)
Median % CpG5 methylation (range)	65.0 (2–98)
Adjacent-to-tumour tissues	*n* = 26
Median % CpG1 methylation (range)	2.0 (0–98)
Median % CpG4 methylation (range)	9.0 (0–57)
Median % CpG5 methylation (range)	28.0 (3–70)
Non-neoplastic breast samples	*n* = 19
Median % CpG1 methylation (range)	5.0 (0–27)
Median % CpG4 methylation (range)	8.0 (0–33)
Median % CpG5 methylation (range)	9.5 (3–30)
Cut-off values (%)	
CpG1	17.5
CpG4	40.0
CpG5	66.5

**Fig. 2:**
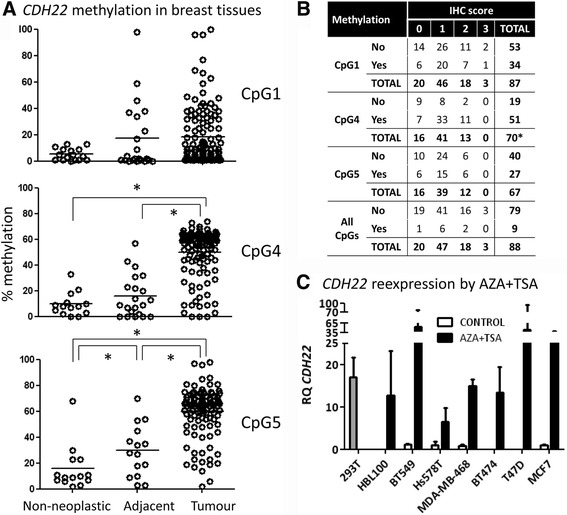
Molecular status of *CDH22* in BC. **a** Methylation in three CpG sites was examined by pyrosequencing in a series of 142 BC cases, along with matched adjacent-to-tumour tissues (*n* = 26), and non-neoplastic mammary tissues from reduction mammoplasties (*n* = 19). The horizontal line in each group represents the median of the series (*p < 0.001). **b** Contingency table showing association between CDH22 immunohistochemical expression and the CpG site methylation status in our series of BC patients (*p = 0.01). **c** *CDH22* expression was restored by epigenetic drugs in six BC cell lines and the immortalised but non-neoplastic mammary cell line HBL-100, as measured by qRT-PCR. 293T cells were used as a positive control

Based on this threshold, higher hypermethylation levels in all CpG sites were observed in tumours than in non-neoplastic tissues, again with intermediate levels in adjacent-to-tumour samples (Fig. 2a). This is the first evidence showing that *CDH22* is epigenetically silenced by promoter hypermethylation in BC.

Next, we interrogated whether *CDH22* promoter methylation levels were correlated with protein expression. Methylation in only the CpG4 site was significantly correlated with immunohistochemical expression (Fig. 2b). However, the statistical significance was lost when considering methylation in all studied CpG sites.

### CDH22 expression can be modulated by epigenetic drugs in BC cell lines

To test whether *CDH22* expression can be dynamically modulated by epigenetic mechanisms, a panel of six BC cell lines and one immortalised but non-neoplastic mammary cell line (HBL-100) were treated with two epigenetic drugs (AZA and TSA). Although a slight decrease in *CDH22* methylation was observed in some cell lines upon treatment with AZA+TSA, a very strong re-expression of *CDH22* mRNA was found by qRT-PCR in all tested cell lines following epigenetic drug treatment (Fig. 2c). These results suggest that epigenetic treatments can restore *CDH22* expression and that this can be dynamically modulated in vitro in BC.

### CDH22 hypermethylation predicts BC progression

**Fig. 3:**
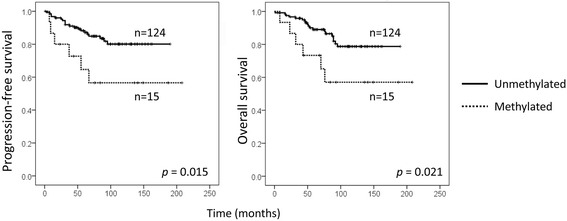
Clinical value of *CDH22* hypermethylation in BC. Significant associations between *CDH22* hypermethylation in all three examined CpG sites and progression-free survival and overall survival were found in our series of BC cases

Lastly, we attempted to examine the clinical value of *CDH22* hypermethylation in our series of 142 BC patients (Table 1). Using the aforementioned cut-off values, we found that *CDH22* hypermethylation in all the 3 CpG sites was significantly associated with shorter progression-free survival (PFS) (*p* = 0.015) and OS (*p* = 0.021) (Fig. 3 and Additional file 4: Figure S4).

Although subtle correlation between protein expression and methylation has been observed in our series, the relationship between immunohistochemical CDH22 protein levels and PFS or OS was examined: no significant association was found between them, although high levels of protein tended to be associated with longer PFS and OS (Additional file 5: Figure S5).

It is well known that several factors, such as BC subtype, lymph node involvement, grade and stage can influence BC prognosis. As expected, these characteristics had an important influence on PFS and OS (Additional file 6: Figure S6). Therefore, the independent impact of *CDH22* hypermethylation on progression and survival, regardless of those clinical variables, was tested in a Cox regression model. It is of particular note that we found that hypermethylation in the *CDH22* promoter was still significantly associated with shorter PFS (*p* = 0.006), irrespective of age and stage (Table 3). The other clinical parameters significantly correlated with PFS and OS (grade and lymph node involvement) were not included in the Cox regression model due to their association with the stage (*p* < 0.001). *CDH22* hypermethylation had a hazard ratio of 4.2 for PFS (Table 3). These results suggest that *CDH22* hypermethylation is an independent predictor of progression in BC.

**Table 3:** *CDH22* hypermethylation as an independent prognostic factor

Variable	PFS		OS	
	Hazard ratio (95% CI)	*p* value	Hazard ratio (95% CI)	*p* value
Age	1.035 (1.005–1.067)	0.021	1.060 (1.026–1.094)	<0.001
Stage	4.149 (1.762–9.772)	0.001	2.450 (0.930–6.453)	0.070
*CDH22* hypermethylation	4.289 (1.507–12.209)	0.006	2.498 (0.821–7.601)	0.107

Cox regression model shows the independent effect of each prognostic factor on progression-free survival (PFS) and overall survival (OS). Stage was divided into two categories: early (stages I, IIA and IIB) and advanced (stages IIIA and IIIC). *CI* confidence interval

## Discussion

This study explored the unknown molecular status and clinical value of CDH22 deregulation in BC, which have been described in other cancer types [16, 17]. We provide the first evidence of the low level of expression of CDH22 in breast tumoral cells compared with non-neoplastic mammary tissue. The exact role of this protein in cancer is controversial. Thus, a lower level of CDH22 protein expression has been reported in metastatic melanoma than in dysplastic nevus [16]. Conversely, mRNA and protein overexpression were described in primary and metastatic colorectal cancer relative to normal mucosa [17]. These observations suggest that the role of CDH22 in cancer development and metastasis is likely to be tissue type-specific [16]. This discrepancy might be explained by the two opposing roles of cell adhesion molecules: to prevent cells from metastasizing by increasing cell-cell adhesion at the site of the primary tumour and to enhance metastatic potential by increasing their anchorage to other cells at distant locations in the body after breaking off from the primary tumour [16, 19]. E-cadherin provides an example of this potential dual tissue-specific role, since it is lost in malignant epithelial cancers, and simultaneously is essential from promoting tumorigenesis in certain cancer types, including ovarian cancer [20] and inflammatory BC [21].

Despite its controversial role, no studies have examined the mechanisms underlying *CDH22* deregulation in cancer. Thus, it has been suggested that mutations, epigenetic silencing and increased proteolysis may be involved in the loss of CDH22 expression [16]. In this study, we have provided the first evidence that CDH22 downregulation in BC relative to non-neoplastic mammary tissues is due to promoter hypermethylation in a subset of cases. Additionally, we have observed that *CDH22* silencing is dynamically restored in vitro by epigenetic drug treatment in a very similar manner in all BC cell lines. This epigenetic alteration has been assessed by pyrosequencing, a technique that yields a quantitative measure of methylation, in contrast to the qualitative technique of methylation-specific PCR [22]. It is worth noting, as reported by other authors [23, 24], that a poly-T-enriched region in this gene promoter has compromised polymerase fidelity, making it difficult to analyse the rest of the gene promoter in several cases.

Importantly, *CDH22* hypermethylation was significantly associated with shorter PFS and OS in our large series of BC patients. Accordingly, *CDH22* deregulation was associated with clinical outcome in other cancer types: loss of CDH22 protein expression was correlated with melanoma progression, and with worse 5-year PFS, and a similar, though not significant pattern, was observed for 5-year OS [16]; in colorectal cancer CDH22 overexpression was significantly and positively correlated with progression, invasion, metastasis and clinical stage of patients [17]. Above all, we showed that *CDH22* hypermethylation, but not expression, was an independent prognostic factor in our BC series. It can predict shorter PFS, regardless of the key factors of age and stage in BC outcome, by using a quantitative and objective method like pyrosequencing in comparison with immunohistochemistry.

## Conclusions

In conclusion, our results show that *CDH22* is hypermethylated in BC, and that this epigenetic alteration is an independent biomarker predicting shorter PFS in BC.

## Methods

### Patient samples

We analysed a series of 142 formalin-fixed, paraffin-embedded samples from BC patients, diagnosed with infiltrating duct carcinoma breast between 1996 and 2006 in the Department of Pathology (Complejo Hospitalario de Navarra, Navarra Public Health System, Pamplona, Spain), upon microscopic evaluation by two independent observers in accordance with the recommended criteria of the St Gallen International Expert Consensus 2013 [4] and considering a Ki-67 threshold of 14% [25], graded according to the Nottingham system [26] and staged with AJCC system [27]. All tumours were surgically removed and staged according to their size, histological grade and degree of lymph node involvement. None of the patients had received radiotherapy or chemotherapy before surgery. Pathological and clinical characteristics are summarised in Table 1. All cases were chosen on the basis of them harbouring at least 70% tumour cells. Additionally, 88 paired non-neoplastic adjacent-to-tumour tissues and 24 non-neoplastic mammary samples from reduction mammoplasties were employed.

### Immunohistochemistry

Three-micrometer sections of 88 BC tumours and their non-neoplastic adjacent-to-tumour counterparts, along with 24 non-neoplastic mammary samples were placed on slides and then deparaffinized, hydrated and treated to block endogenous peroxidase activity. After incubating with the primary rabbit polyclonal CDH22 antibody (ab171616, Abcam, Cambridge, UK) at 1:100 dilution for 20 min (antigen retrieval at 90 °C for 20 min, pH = 6.0), the antibody was developed using a Bond Polymer Refine Detection kit (Leica, Wetzlar, Germany) and visualised with diaminobenzidine. The pattern of expression was blind-evaluated by two independent observers. The intensity of expression was ascribed to one of four categories: 0, no expression; 1, weak expression; 2, intermediate expression; and 3, strong expression. Images were acquired with a Leica DM 4000B microscope (Leica, Wetzlar, Germany).

### Cell lines and treatments

A panel of six human BC cell lines (T-47D, BT-474, BT-549, MDA-MB-468, Hs 578 T and MCF-7) and one immortalised but non-neoplastic mammary epithelial cell line (HBL-100) were used in this study. T-47D, BT-474, BT-549 and HBL-100 cell lines were purchased from the American Type Cell Collection (ATCC, Rockville, MD, USA). MDA-MB-468 and MCF-7 cell lines were obtained from the Leibniz Institute DSMZ-German Collection of Microorganisms and Cell Cultures (Braunschweig, Germany). The Hs 578T cell line was kindly provided by Dr Javier Benítez (Human Genetics Group, Spanish National Cancer Research Centre, Madrid, Spain). The human embryonic kidney 293T cells (ATCC, Rockville, MD, USA) were used as a positive control for *CDH22* expression. All cell lines used were grown in RPMI-1640 or DMEM supplemented with 10% foetal bovine serum and 1% penicillin/streptomycin (all from Life Technologies, Carlsbad, CA, USA), at 37 °C in a humidified atmosphere with 5% CO_2_.

BC cell lines were treated with two epigenetic drugs: the demethylating agent 5-aza-2′-deoxycytidine (AZA) and the histone deacetylase inhibitor trichostatin A (TSA) (both from Sigma-Aldrich, St Louis, MO, USA). Briefly, cells were seeded at a density of 1 × 10^5^ cells/ml, allowed to attach overnight and treated with 4 μM AZA for 72 h by adding the drug every 24 h, 300 nM TSA for 24 h or the combination of both drugs for the last 24 h, using PBS as the vehicle control.

### DNA extraction, bisulphite conversion and pyrosequencing

To determine the methylation status of the *CDH22* gene, DNA was extracted from formalin-fixed, paraffin-embedded breast tumours, adjacent-to-tumour tissues and non-neoplastic mammary tissues using a QIAamp DNA FFPE Tissue kit (Qiagen, Hilden, Germany). Bisulphite conversion of DNA was performed to transform unmethylated cytosines into thymidines, while methylated cytosines remained intact. Five hundred nanograms of DNA were treated with freshly prepared bisulphite using the EZ DNA Methylation-Gold kit (Zymo Research, Irvine, CA, USA) in accordance with the manufacturer’s recommendations. Pyrosequencing was carried out to analyse the methylation of five CpG sites in the promoter of the *CDH22* gene (Additional file 1: Figure S1). For this purpose, first, PCR amplification was performed using Immolase DNA polymerase (BioLine, London, UK) in a final volume of 30 μl containing 2 μl of bisulphite-modified DNA and two sets of primers (i) forward primer 5′-GGTTTTTGATGGAAAGGGAAGGTTTTTA-3′, reverse primer 5′-BIOTIN-CCAAACAACACCTAAACAACTCCAAAAT-3′, (ii) forward primer 5′-BIOTIN-GGTTTTTGATGGAAAGGGAAGGTTTTTA-3′, reverse primer 5′-CCAAACAACACCTAAACAACTCCAAAAT-3′). Amplification conditions were initial DNA polymerase activation at 95 °C for 10 min followed by 50 cycles at 95 °C for 30 s, 67 °C for 30 s and 72 °C for 30 s, and final extension at 72 °C for 7 min. The amplicons were resolved by electrophoresis using 2% (*w*/*v*) agarose gel in 1 × tris-borate-EDTA buffer, stained using SYBR Red Safe (Life Technologies, Carlsbad, CA, USA) and visualised in a standard transilluminator (ChemiDoc XRS, Bio-Rad Laboratories, Hercules, CA, USA). Quantitative DNA methylation analysis was done as follows: 20 μl of PCR products were immobilised with Streptavidin Sepharose HP Beads (GE Healthcare Bio-Sciences, Pittsburgh, PA, USA) using a Vacuum Prep Work station. Two different sequencing primers (one forward 5′-GTTTTTAGTTTTGGTAGGAT-3′ for the amplicons generated with the first set of PCR primers and one reverse 5′-ACACCTAAACAACTCCA-3′ for the amplicons of the second set of PCR primers) were then annealed at 80 °C for 2 min in different reactions and pyrosequenced in a PyroMark Q24 using PyroMark Gold Q24 reagents and PyroQ-CpG^TM^ Software (v.1.0.11) (all from Qiagen, Hilden, Germany). Results were analysed with PyroMark Q24 software in CpG analysis mode. Only methylation values found to be of high quality were considered.

### RNA extraction and quantitative reverse transcription PCR

Quantitative reverse transcription PCR (qRT-PCR) was performed to assess the restoration of *CDH22* expression by AZA+TSA treatment in BC cell lines. Briefly, total RNA was extracted and purified using an RNeasy Mini Kit (Qiagen, Hilden, Germany) following the manufacturer’s instructions. Five hundred nanograms of total RNA were retrotranscribed using a PrimeScript™ RT reagent Kit (TaKaRa, Otsu, Japan) under conditions of 37 °C for 15 min and 85 °C for 5 s. One microliter of the resulting cDNA was placed in a 96-well plate with 0.5 μl TaqMan probes (*CDH22*, Hs.PT.58.50475831; *GAPDH*, Hs.PT.58.40035104 and *ACTB*, Hs.PT.39a.22214847 from IDT, Coralville, Iowa, USA; and *18S*, Hs99999901_s1, from Life Technologies, Carlsbad, CA, USA) and 19 μl of the Premix Ex Taq™ kit (TaKaRa, Otsu, Japan). PCR amplification was performed in triplicate using the Quant Studio 12 K Flex (Life Technologies, Carlsbad, CA, USA) under thermal cycler conditions of 95 °C for 30 s and 40 cycles at 95 °C for 5 s and 60 °C for 34 s. The cycle threshold (Ct) values were calculated using Quant Studio software (Life Technologies, Carlsbad, CA, USA). The relative quantification (RQ) was calculated following the ΔCt method (RQ = 2^−ΔCt^), using *GAPDH*, *ACTB* or *18S* as the endogenous control genes. Among them, *GAPDH* was found to be the better endogenous gene in our cell lines, with a smaller coefficient of variation. Therefore, relative expression of *CDH22* was normalised with respect to the level of *GAPDH* expression.

### Statistical analysis

Demographic, clinical and pathological data were summarised as frequencies (and percentages) or means and medians (and ranges), as appropriate. The differences in the frequency of immunohistochemical expression in non-neoplastic, adjacent-to-tumour, and tumour groups were evaluated by the Fisher’s exact test. The optimal cut-off value identifying the methylated or unmethylated status of the *CDH22* gene promoter and predicting PFS and OS was estimated by ROC curve analysis, as previously described [28]. Statistical differences in CpG site methylation between groups were determined by Mann–Whitney’s test. Kaplan–Meier plots and log-rank tests were used to examine the association between *CDH22* methylation or expression and PFS and OS. A multivariate Cox regression model was fitted to test the independent contribution of each variable to the patient’s outcome after adjustment. Hazard ratios and 95% confidence intervals were used to estimate the effect of each variable on the outcome. Associations between clinical variables were tested by the *χ* ^2^ test.

## Additional files

Additional file 1: Figure S1. The *CDH22* gene promoter. Bisulphite-converted sequence of the *CDH22* promoter, highlighting the five CpG sites studied. (TIF 570 kb)
Additional file 2: Figure S2. Representative pyrograms of the *CDH22* promoter in breast tissues. Pyrosequencing was conducted with two sequencing primers to obtain high quality results of methylation percentages in more CpG sites. Blue, yellow and red boxes indicate high, acceptable and unacceptable quality results. (TIF 1863 kb)
Additional file 3: Figure S3. Cut-off value for *CDH22* methylation. ROC curve analysis was used to estimate the optimal cut-off values of each of the CpG site methylation able to distinguish the unmethylated or methylated status of the *CDH22* gene promoter. Here ROC curves for the CpG1 site are shown. (TIF 407 kb)
Additional file 4: Figure S4. Association between individual CpG site hypermethylation and clinical parameters in BC. Among the three CpG sites analysed, the hypermethylation only of the CpG1 was found to be statistically associated with a poor progression-free survival and shorter overall survival. (TIF 392 kb)
Additional file 5: Figure S5. Clinical value of CDH22 protein expression in BC. Associations between CDH22 protein levels and progression-free survival and overall survival were examined in our series of 88 BC cases. (TIF 273 kb)
Additional file 6: Figure S6. Clinical value of factors of importance in BC. Associations between progression-free or overall survival and BC subtype (LA, luminal A; LB, luminal B/HER2-negative; LH, luminal B/HER2-positive; H, HER2; TN, triple-negative), lymph node involvement, histological grade and stage were analysed in our series of 142 BC patients. (TIF 521 kb)
