# Supplementary figures and images for: CDH22 hypermethylation is an independent prognostic biomarker in breast cancer
Source: Clin Epigenetics. 2017 Jan 24;9:7. doi: 10.1186/s13148-016-0309-z (PMC5270318; doi:10.1186/s13148-016-0309-z)

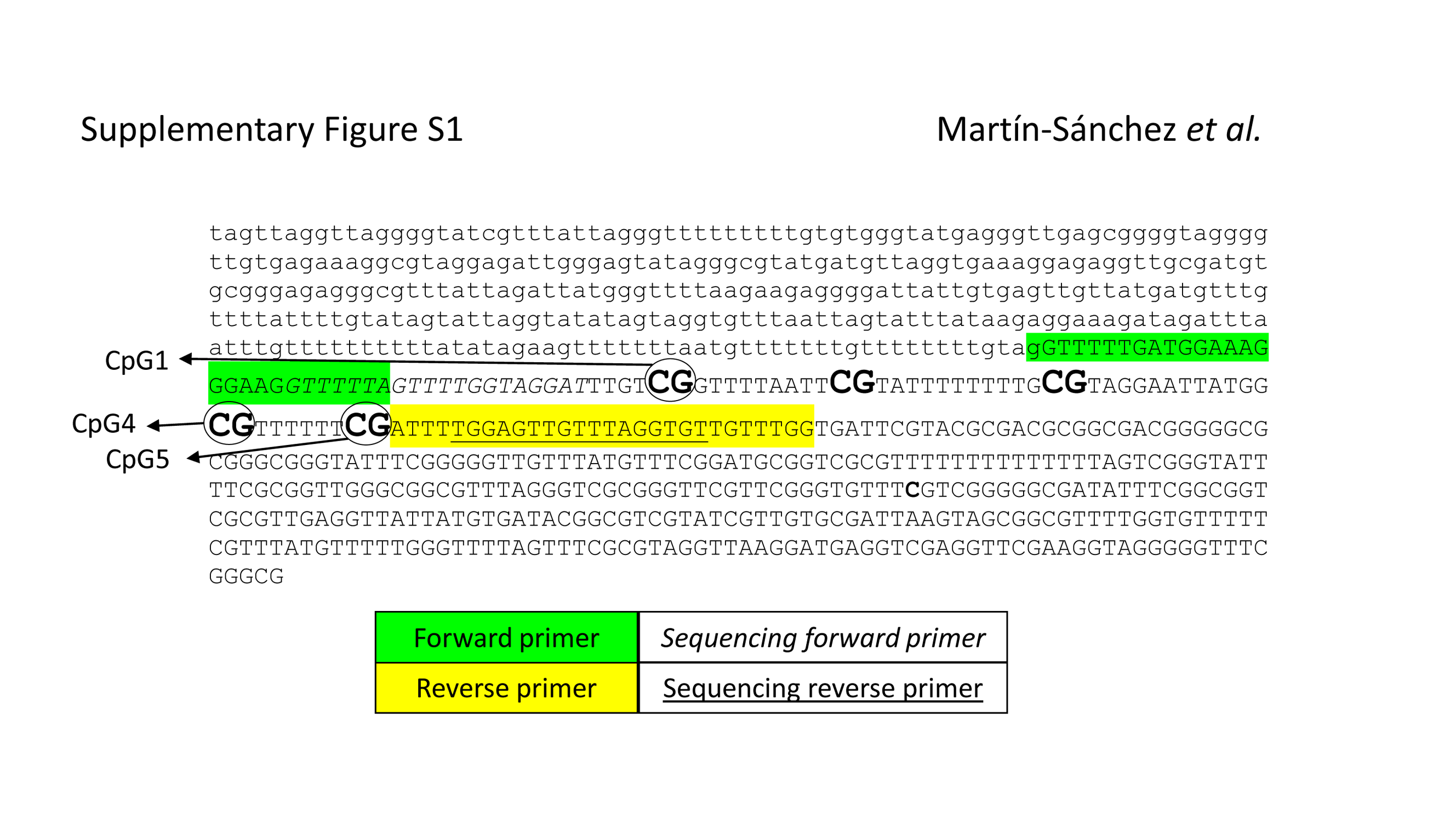

Supplement: Additional file 1: Figure S1. — The CDH22 gene promoter. Bisulphite-converted sequence of the CDH22 promoter, highlighting the five CpG sites studied. (TIF 570 kb) [file 13148_2016_309_MOESM1_ESM.tif]

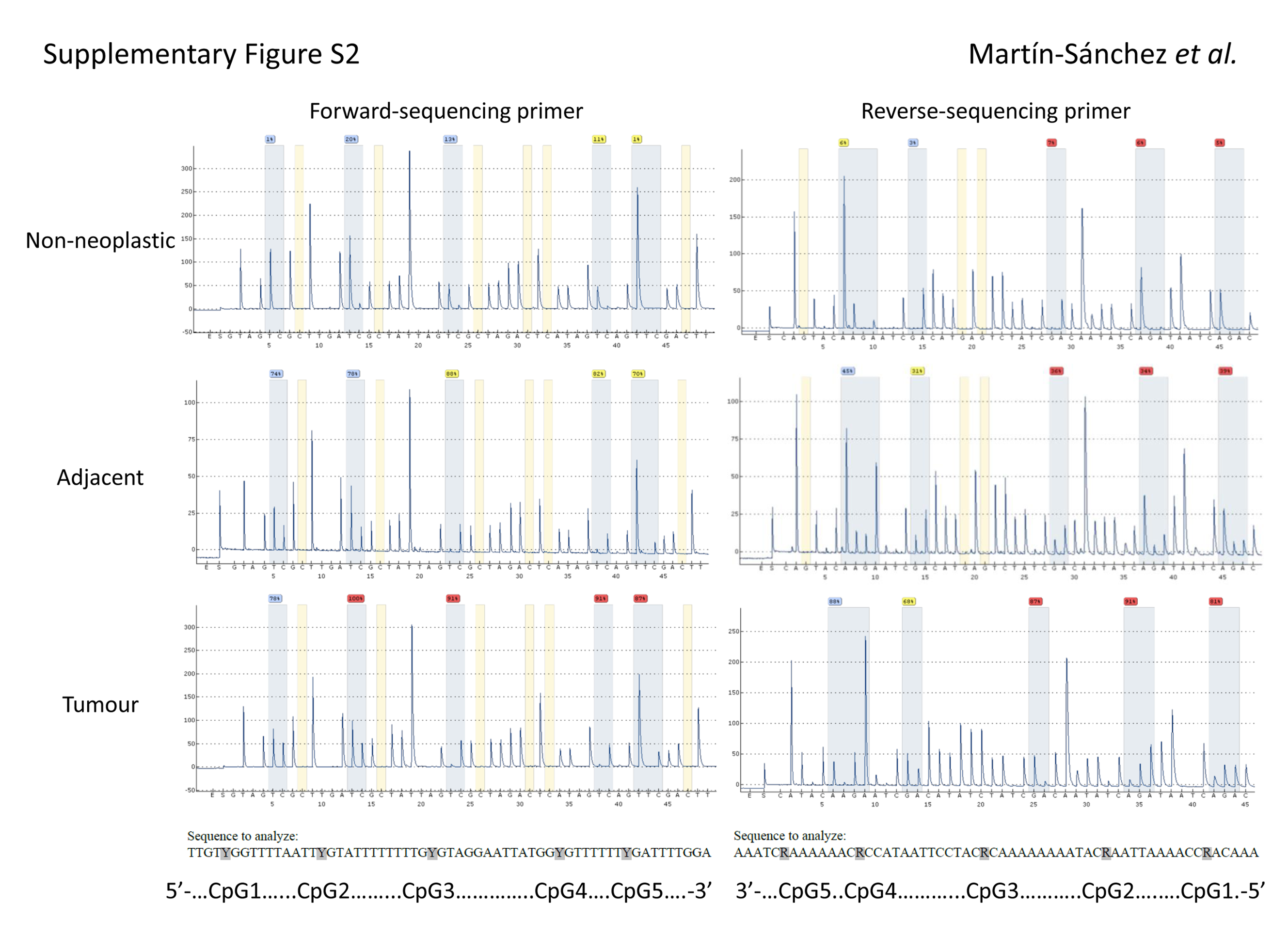

Supplement: Additional file 2: Figure S2. — Representative pyrograms of the CDH22 promoter in breast tissues. Pyrosequencing was conducted with two sequencing primers to obtain high quality results of methylation percentages in more CpG sites. Blue, yellow and red boxes indicate high, acceptable and unacceptable quality results. (TIF 1863 kb) [file 13148_2016_309_MOESM2_ESM.tif]

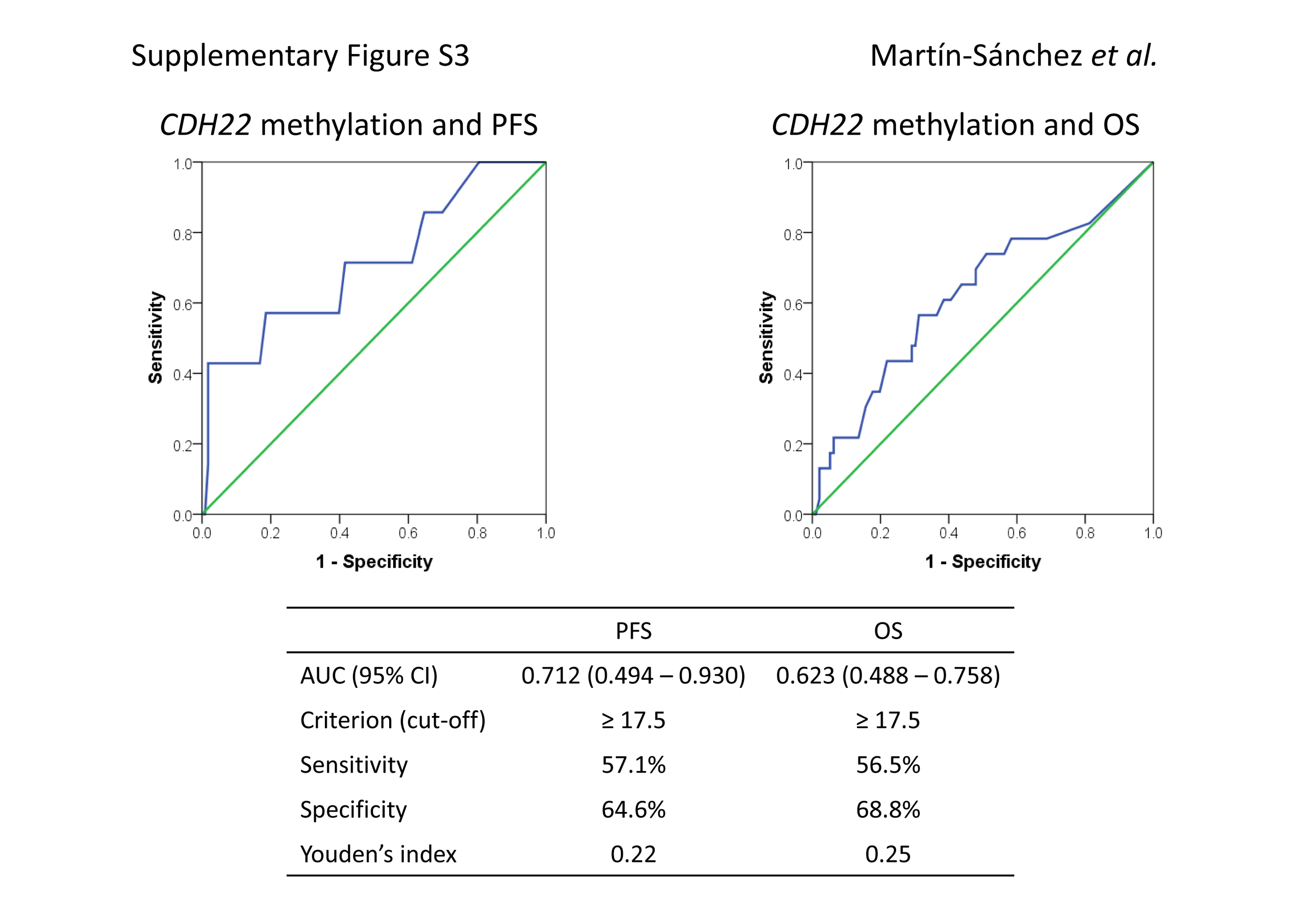

Supplement: Additional file 3: Figure S3. — Cut-off value for CDH22 methylation. ROC curve analysis was used to estimate the optimal cut-off values of each of the CpG site methylation able to distinguish the unmethylated or methylated status of the CDH22 gene promoter. Here ROC curves for the CpG1 site are shown. (TIF 407 kb) [file 13148_2016_309_MOESM3_ESM.tif]

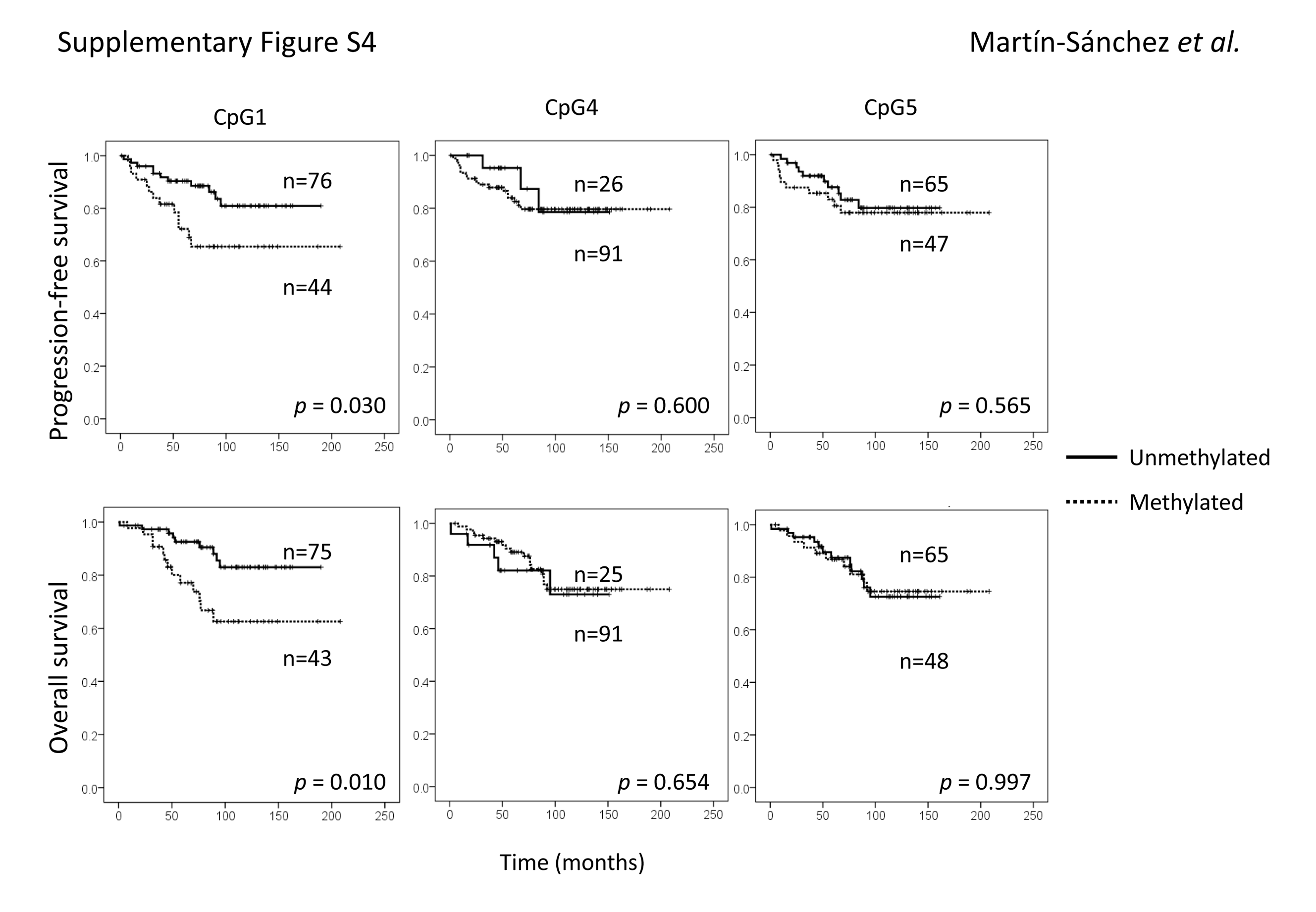

Supplement: Additional file 4: Figure S4. — Association between individual CpG site hypermethylation and clinical parameters in BC. Among the three CpG sites analysed, the hypermethylation only of the CpG1 was found to be statistically associated with a poor progression-free survival and shorter overall survival. (TIF 392 kb) [file 13148_2016_309_MOESM4_ESM.tif]

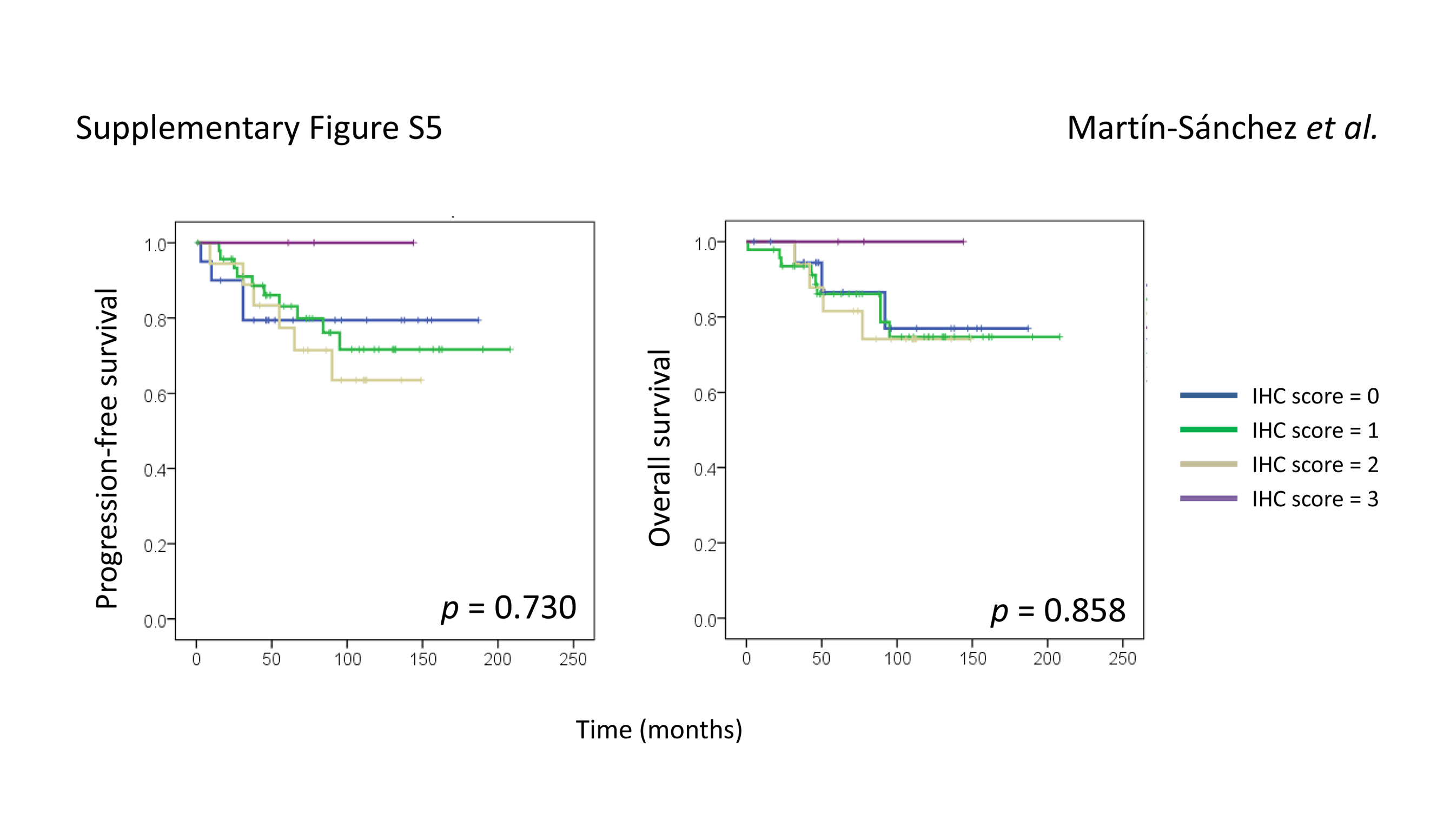

Supplement: Additional file 5: Figure S5. — Clinical value of CDH22 protein expression in BC. Associations between CDH22 protein levels and progression-free survival and overall survival were examined in our series of 88 BC cases. (TIF 273 kb) [file 13148_2016_309_MOESM5_ESM.tif]

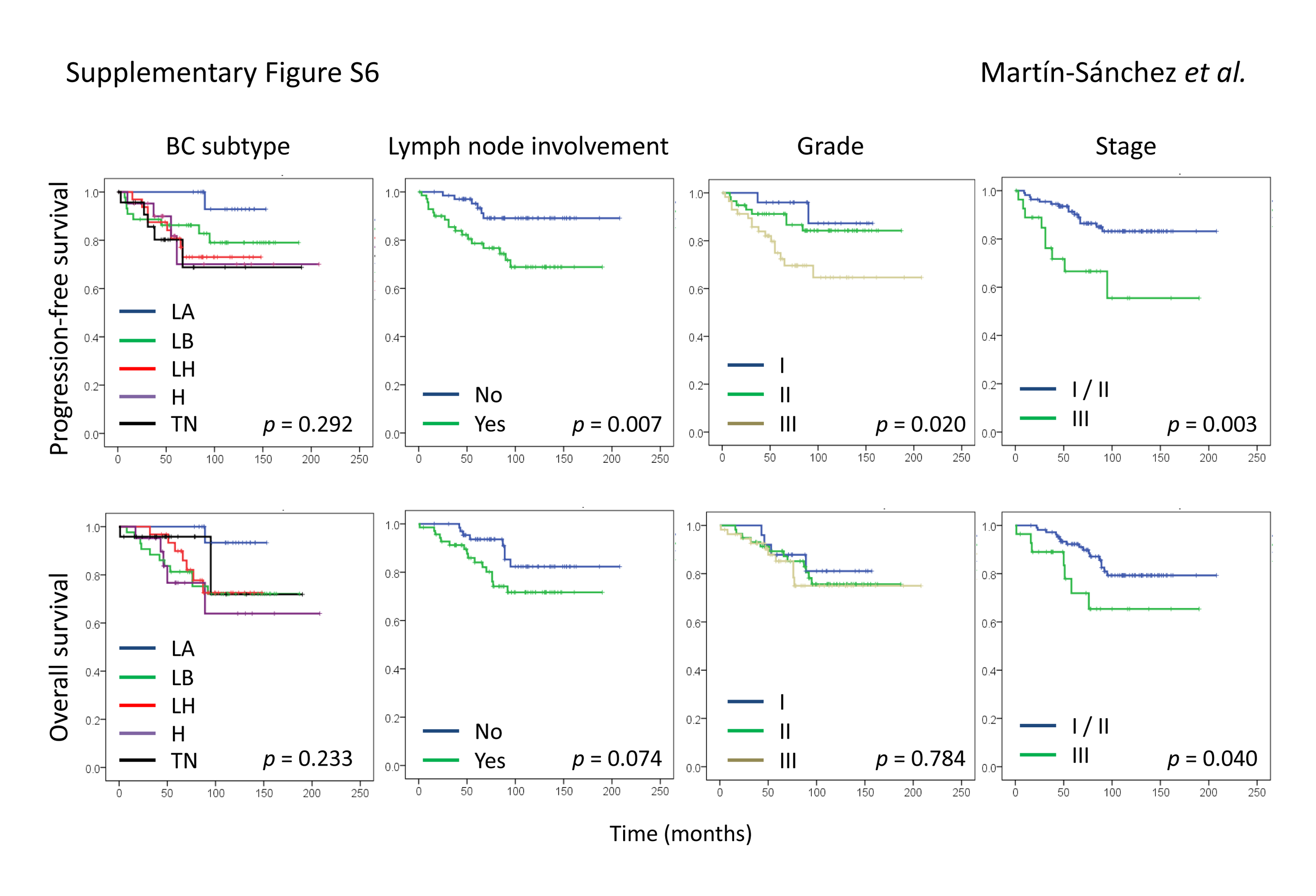

Supplement: Additional file 6: Figure S6. — Clinical value of factors of importance in BC. Associations between progression-free or overall survival and BC subtype (LA, luminal A; LB, luminal B/HER2-negative; LH, luminal B/HER2-positive; H, HER2; TN, triple-negative), lymph node involvement, histological grade and stage were analysed in our series of 142 BC patients. (TIF 521 kb) [file 13148_2016_309_MOESM6_ESM.tif]
